# Supplementary material for: Are carbon emissions trading and green financial instruments synergistic? -Comprehensive quantitative research based on content analysis
Source: PLoS One. 2024 Mar 7;19(3):e0298601. doi: 10.1371/journal.pone.0298601 (PMC10919601; doi:10.1371/journal.pone.0298601)
Supplement: S1 Data — (ZIP) [file pone.0298601.s001.zip › Data/地级市Z_F工作报告.zip!/╜¡╦╒/╒≥╜¡╩╨/└┤╫╘╣1⁄2╓┌║┼í╛┬φ┐╦╩2╛▌═°í┐_2006.docx]

各位代表：

　　从今年开始，镇江步入了“十一五”发展新时期。全力打造新镇江，努力让镇江发展得更快更好，让镇江人民更加富裕安康，是政府的神圣职责。现在，我代表镇江市人民政府，向大会作工作报告，请予审议，并请市政协各位委员和其他列席人员提出意见。

　　一、2005年和“十五”回顾

　　2005年，在中共镇江市委的领导下，全市上下深入贯彻科学发展观，按照“全面提升、全力争先”的总体要求，创新克难，锐意进取，全面完成市五届人大三次会议确定的各项目标任务，为“十五”发展划上了圆满句号。

　　（一）国民经济健康发展

　　完成地区生产总值905亿元，增长15％，人均达到4150美元。财政总收入118.4亿元，增长26.6％，地方财政一般预算收入47.1亿元，增长26.4％。全社会固定资产投资400.1亿元，增长25％。城镇居民人均可支配收入12394元，农民人均纯收入6020元，分别增长14.1％和13.5％。社会消费品零售总额220.6亿元，增长15.2％。

　　农业基础地位加强。粮食生产克服灾害影响，实现总产90.7万吨。实施“放心粮（油）、放心肉、放心菜”工程，新增无公害农产品产地面积25万亩。“三资”开发农业项目440个，总投资33亿元。全力抓好禽流感等重大动植物疫病防控。新建、改造塘坝994座，疏浚县乡河道78条，治理水土流失面积11.3平方公里。新增耕地近1.6万亩，完成中低产田改造5.75万亩，成片造林3.1万亩。

　　工业运行平稳。规模以上工业增加值完成358.4亿元，增长23.5％。实现销售收入1231亿元、利税95.9亿元，分别增长25.3％和24.9％。完成工业性投入235.1亿元，增长30.3％。积极培育规模企业，索普集团、大亚科技、大全集团、中电电气、鼎胜铝业等企业增势强劲。新增销售超亿元企业40家。积极推进名牌战略，新增全国驰名商标1个、中国名牌2个、国家免检产品5个。

　　服务业发展迅速。完成增加值322.3亿元，增长16.6％。旅游市场持续升温，旅游业增加值占GDP的比重提高到6.5％。新型商贸业态不断涌现，天润发、沃尔玛等知名企业相继落户，长江新天地、农副产品批发市场、润扬国际工业物流中心、金太阳家居广场等一批重点项目开工建设。金融机构新增人民币贷款88.94亿元。金融资产质量明显提高，对经济的支持力度进一步加大。商品房销售面积154.8万平方米。

　　（二）群众生活继续改善

　　为民办15件实事全面完成。新增城镇就业2.55万人，转移农村劳动力6.52万人。城镇登记失业率3.5％，处于全省最低控制线以下。发放《再就业优惠证》10500本，近8000人实现再就业。完善工资宏观指导机制，最低月工资标准由500元提高到550元。城镇职工平均工资增长13.8％，农民工资性收入增长18.7％。社会保障不断完善。养老保险基金连续三年实现收支硬平衡，离退休人员养老金继续保持100％社会化发放。全市社会医疗保险参保人数超过210万人，城镇职工参保率99％以上；城乡居民合作医疗保险覆盖率达91.6％。4万多名被征地农民进入基本生活保障体系。成立市慈善总会，募集资金3385万元。城市低保标准由200元提高到230元，初步建立城乡一体的医疗救助制度，已有5000名城乡困难人员进入医疗救助体系。扩大市区住房保障覆盖面，新增住房保障家庭181户。茅山老区扶贫工作取得新成绩。

　　（三）改革开放不断深入

　　体制改革稳步推进。国有资产管理体制改革继续完善。市属中小企业改制基本完成，索普集团等大型企业改制顺利实施。生产经营性事业单位改革稳步推进。市直国库集中收付制度改革全面推开。新一轮乡镇行政区划调整基本完成。土地市场健康发展，完成市区土地级别更新，出让经营性用地2207亩，土地成交金额14.92亿元。

　　民营经济加快发展。全市新增私营企业3000家、个体工商户1.5万户，新增注册资本40亿元，实际吸纳民资122亿元。规模以上工业民营企业完成增加值147.5亿元，增长24.3％，占全市规模以上工业比重为41％。

　　对外开放态势良好。完成合同利用外资32.4亿美元，实际到位外资9.2亿美元（原口径），分别增长20.9％和51.8％。完成进出口总额39.1亿美元，增长12.5％，其中出口20.3亿美元，增长33.6％。外经完成营业额1.62亿美元，新批境外投资企业9家。全市开发园区基础设施投入26.8亿元，增长21％，园区产业集聚效应进一步显现。以大港三期工程为重点，加强港口建设，开工建设泊位13个，其中万吨级5个。完成外贸运量1000万吨、港口吞吐量5800万吨，分别增长25％和20％。外事、侨务、对台工作取得新进展，与德国曼海姆市结为国际友好城市。

　　（四）城市建设全力推进

　　城市功能提升。润扬大桥建成通车，沪宁高速、312国道镇江段拓宽改造完成。建成金桥大道、中山北路、纬六路、宗泽路、通港北路等城市干道。改造市区10个老住宅片区，总面积55万平方米；改造街巷道路80条；治理危房1万平方米。解决部分积水区内涝问题，完成市区4座山体滑坡综合治理。加快天然气工程建设，12万户居民用上天然气。新增环保节能公交车63辆，新辟、调整优化公交线路9条；三年投资10多亿元，全面完成农村公路工程，行政村班车通车率达96％。

　　生态建设加强。生态示范区创建工作扎实推进。句容、扬中创建国家卫生城市、国家环保模范城市取得实质性进展。市区新增绿地120万平方米，成功创建为国家园林城市。高度重视饮用水安全，颁布并实施征润洲和黄岗饮用水水源地保护方案。加快建设城东垃圾填埋场和无害化粪便处理场，完成丹徒新区污水处理厂一期主体工程。开展循环经济试点工作，30家企业通过清洁生产审核。企业环境行为信息公开化制度继续走在全国前列。关闭违法排污企业25家；对钛白粉厂实施搬迁，上铁水泥厂停产拆除；关闭非法开采矿山企业19家、采石宕口32个。全年空气质量良好以上天数达322天。

　　城管水平提高。新一轮城市总体规划在省内率先编制完成并开始实施。加强主次干道20小时保洁和街巷居民小区“三位一体”保洁，18条新建城市道路保洁权实行市场化运作，城市长效管理机制初步形成。坚持以人为本、有情操作，成功开展“三小车”整治，一举解决这一困扰城市管理多年的难题，市容环境和交通秩序明显改观。

　　（五）社会事业加快发展

　　大力推进“双倍增”计划，高新技术产业增加值占规模以上工业增加值的25.8％，比上年提高1.8个百分点；新认定省级以上高新技术企业26家、省级高新技术产品68项。重大科技成果转化成效显著，7个项目被列入省专项，获拨款资助超亿元，居全省第一。专利申请量超过1800件。被国家知识产权局列为“全国知识产权试点城市”。“水环境质量改善与生态修复技术研究及示范”项目按序时进度推进。引进培养高层次人才1574人。初中毕业生升学率连续三年保持在95％以上，高中阶段毛入学率达到85.3％，适龄青年接受高等教育比例达47％。92％的乡镇通过“实施江苏省教育现代化工程示范（先进）乡镇”评估验收。区域卫生发展规划顺利实施，城区80％以上街道、辖市所有街道和85％以上乡镇建成社区卫生服务中心。市疾控中心和卫生监督所综合业务大楼、市传染病医院一期工程按计划实施，市儿童医院落成。农村自来水普及率达98％以上。建成全民健身工程（点）226个。镇江博物馆新馆开馆，并被国家旅游局批准为4A级风景旅游区；苏南抗战胜利纪念碑二期工程竣工。成功承办十运会女排和男篮部分比赛，镇江籍运动员在十运会上获6金2银1铜的佳绩。完成第一次全国经济普查、人口抽样调查任务。落实双拥政策，完成军地互办20件实事。发放农村部分计划生育家庭奖励扶助金315万元。

　　（六）民主法制和政府自身建设得到加强

　　社区居委会和新一届村委会换届工作基本完成。“平安镇江”创建活动不断深入，“打防控”格局基本形成，城乡治安良好、社会稳定。出台反恐怖袭击及突发事件处置意见和重特大生产安全事故应急救援预案，安全生产形势总体平稳，工矿商贸企业未发生重大安全事故。坚持和完善领导干部信访接待日制度，建立健全社会矛盾纠纷调处网络，加大处理信访突出问题的力度。自觉接受市人大及其常委会和市政协监督，办理人大代表建议232件、政协提案467件，代表、委员的满意和基本满意率分别为98％、96.6％。不断加强依法行政工作，加快“法治政府”建设。行政审批制度改革进一步深化，再次取消审批事项105项。市政府门户网站“中国镇江”服务功能达到国内先进水平，各组成部门全面开通电子政务网站。加强廉政建设。成立市行政效能监察中心，加大行政监察和责任追究力度。开设“行风热线”，妥善解决群众反映的各类问题。市行政服务中心建设继续加强，34个部门基本完成“两集中、两到位”，即办件占中心日均办件量的54％，承诺件的平?事务后勤保障有力。

　　各位代表，“十五”时期是我市发展历程上十分重要、很不平凡的时期。经过全市上下的共同努力，镇江发生了巨大变化！

　　过去的五年，是率先发展的五年，镇江综合实力显著增强。地区生产总值及人均数实现翻番，年均分别增长13.3％和13.1％。财政总收入增长2.6倍。累计完成投资1300亿元，其中电力、港口等基础产业投入80亿元。农业产业化水平明显提升，旅游、物流等新型服务业蓬勃发展。工业产业集中度显著提高，机械、化工、造纸主导产业地位进一步确立，涌现出一批新兴产业集群。

　　过去的五年，是开拓创新的五年，镇江经济活力显著提高。基本完成国有企业改制和农村税费改革，事业单位改革全面展开。累计实际到位外资37.7亿美元，超过此前历年总和。“十五”期末外贸出口是“九五”期末的3倍，镇江口岸与全球近一半的国家和地区开通贸易往来。民营经济占到全市经济总量的“半壁江山”。

　　过去的五年，是加快建设的五年，镇江城乡面貌显著变化。对外交通和城市建设投入累计超过310亿元，拉开了城市框架，扩大了城市规模。新增道路面积590万平方米，新增公共绿地面积300万平方米，城市化水平达58％。荣获国家卫生城市、国家环保模范城市等称号。辖市（区）主城区和中心镇建设加强，农村“五件实事”圆满完成。

　　过去的五年，是富民优先的五年，镇江人民生活显著改善。城镇居民人均可支配收入和农民人均纯收入年均分别增长11.6％和8.3％。2005年，居民储蓄存款余额458.5亿元，比“九五”期末增加254.5亿元。遍及城乡的社会保障体系初步建立。市区城镇居民人均住房建筑面积23.9平方米。

　　过去的五年，是协调推进的五年，镇江社会事业显著进步。连续五届荣获“全国科技进步先进市”称号，教育现代化步伐加快，涌现出一批文化创作精品。全民健身工程遍布城乡，医疗卫生条件明显改善。“平安镇江”、“法治镇江”创建活动深入开展，成为“全国社会治安综合治理优秀地市”。精神文明建设不断加强，新时期“镇江精神”得到弘扬，市民素质和城市文明程度进一步提高。

　　各位代表，镇江“十五”时期取得的巨大成绩，来之不易，令人鼓舞。在困难和矛盾面前，全市人民振奋精神、锐意拼搏，积极应对宏观调控，成功战胜非典疫情，完成集资兑付30亿元，镇江获得了稳健发展！在竞争和挑战面前，全市人民争创一流、奋发作为，认真贯彻科学发展观，抢抓跻身苏南板块新机遇，咬定“两个率先”新目标，镇江的发展充满活力！对全市广大干部群众和各界人士在这五年中所付出的辛劳和智慧，我和市政府全体成员都深切感到，用“感谢”二字远远不能表达我们的敬意。镇江人民和所有支持镇江发展的人士在“两个率先”征程中所作出的努力，必将载入史册，并成为我们进一步做好政府工作的强大动力！

　　奋斗的历程难以忘怀，发展的经验弥足珍贵。在工作中，我们真切体会到，坚持以人为本、富民优先，任何时候都不能忘记；坚持率先发展、科学发展，任何时候都不能动摇；坚持开拓奋进、创新克难，任何时候都不能削弱；坚持求真务实、真抓实干，任何时候都不能懈怠。在肯定成绩、总结经验的同时，我们也清醒看到，镇江在前进的过程中还存在一些矛盾和问题。主要是：经济增长方式还比较粗放，产业层次还不够高，经济结构性矛盾没有得到根本解决；城乡居民就业压力较大，富裕程度还不高，农民增收的长效机制尚未形成；征地拆迁、企业改制、转岗就业还存在一些矛盾，社会不稳定因素还比较多；政府职能转变还不能完全适应市场经济的要求，发展环境有待进一步优化。对这些问题，我们将高度重视，务实解决。

　　二、“十一五”发展展望

　　未来五年，是我市实现“两个率先”的关键五年。编制好“十一五”规划，谋划好“十一五”发展，全市关注，责任重大。根据市委四届十二次全会审议通过的《关于制定镇江市国民经济和社会发展第十一个五年规划的建议》，市政府编制了《镇江市国民经济和社会发展第十一个五年规划纲要（草案）》，提请大会审议。

　　“十一五”期间，镇江发展的总体要求是：高举邓小平理论和“三个代表”重要思想伟大旗帜，以科学发展观统领经济社会发展全局，紧紧围绕“两率先、两步走”的奋斗目标，以转变增长方式、统筹城乡发展、构建和谐社会为战略重点，以深化改革、扩大开放和自主创新为基本动力，坚定不移地走富民优先、集聚吸纳、改革创新、特色发展、和谐文明之路，全力打造以人为本、全面协调可持续发展的新镇江。

　　经过五年的奋斗，努力实现以下几个方面的目标：

　　经济发展跃上新台阶。到2010年，地区生产总值力争翻一番，年均增长13.5％以上；财政总收入翻一番，地方财政一般预算收入突破100亿元；规模以上工业销售收入翻一番，突破2500亿元；全社会固定资产投资五年累计3400亿元，年均增长18％；工商注册实际到位外资年均增长20％，五年累计达50亿美元，自营出口年均增长18％，期末达到45亿美元；民营经济增加值占地区生产总值比重超过60％；社会消费品零售总额年均增长14％；单位地区生产总值能耗比“十五”期末降低20％左右。

　　人民生活得到新提高。到2010年，力争城镇居民人均可支配收入、农民人均纯收入分别达到22000元和10000元。消费结构升级，文教娱乐及服务支出占家庭消费支出比重达到18％，恩格尔系数低于38％。社会保障体系比较健全，城镇养老、医疗、失业保险覆盖率均达98％以上，新型农村合作医疗和农村养老保险覆盖面分别达95％和80％以上。城镇登记失业率控制在4％以内。

　　城市建设再上新水平。南徐新城初具规模，北部滨水区初展风采，世业洲旅游度假区初见雏形。在充分保持历史街区风貌的基础上，对老城区进行保护性改造。城市功能不断完善，城市现代化水平明显提高。到2010年，城市化率达到65％左右，城市建成区绿化覆盖率达40％以上，全市环境质量综合指数达90。

　　社会发展开创新局面。到2010年，全社会R&D（研发）投入占到GDP的2％，高新技术产品销售收入超1000亿元。全面推进素质教育，率先基本实现教育现代化。卫生服务体系健全率达到95％，城乡公共文体服务设施明显改善。努力建设学习型城市、创新型社会，争创全国文明城市。民主法制建设更加完善，政府依法行政水平明显提高，市民法律意识显著增强，基本形成具有较高和谐程度的社会发展体系。

　　各位代表，未来五年，将是镇江大发展、大变化、大提升的五年。一个崭新的镇江，将在经济社会发展的各个领域发生可喜变化：

　　——“南山北水、一体两翼”的城市新格局基本形成，镇江山水灵气充分释放，人文底蕴充分发掘，现代气息充分彰显，成为长三角独具特色的历史文化名城、港口型经济城市和滨江山林城市。

　　——镇泰过江通道、高速公路联网、城际轨道交通等重大桥梁、道路工程开工建设，镇江对外交通更加便捷、畅达。

　　——机械、化工、造纸等主导产业规模质量进一步提升，电子信息、新材料、电力、交通设备、食品五大优势产业发展迅猛，汽摩零部件等十大产业集群特色明显，一批大企业、大集团在国内外市场崭露头角。

　　——沿江开发取得全面突破，通过加大投入、加快整合，到2010年，镇江港成为亿吨大港。

　　——城乡统筹步伐加快，社会更加和谐，九年制义务教育实行免费，城乡教育均衡发展，文化体育会展中心等重要文体设施建成，社会主义新农村建设初见成效。

　　放眼未来，审时度势，我们仍将面临更多挑战、更多考验，必须始终把握好以下战略要点：

　　一是必须牢牢坚持“率先发展、科学发展、和谐发展、特色发展”的战略导向。更加自觉地全面贯彻落实科学发展观，认清发展形势，研究发展规律，把握发展要求，实现更快更好发展。坚定不移地做强工业经济、开放型经济、港口型经济、县区经济和主导产业。

　　二是必须始终咬定“两率先、两步走”的战略目标。面对苏南强市快速发展的压力和苏中沿江城市奋力赶超的挑战，要增强忧患意识、危机意识、责任意识，高扬“两个率先”的旗帜，按照既定的序时进度，狠抓落实，狠抓推进。

　　三是必须认真落实“富民优先、科教优先、环保优先、节约优先”的战略方针。坚持以富民的水平检验经济发展的水平，以科技教育的先进程度来衡量社会发展的程度，以环境保护的高水准确保发展的高质量，以节约发展的效率提升科学发展的效能，着力使镇江的发展经得起人民的检验、时代的考验和后人的评价。

　　四吸纳、城乡统筹”等战略举措。推进“十一五”发展，要突出抓大事、抓重点、抓关键。在自主创新上，着眼于“高”，抢占制高点，实现高新化；在结构调整上，着眼于“优”，培育优势产业、优势企业、优势产品；在集聚吸纳上，着眼于“大”，吸引优质大项目，实施有效大投入；在城乡统筹上，着眼于“统”，统一规划，统筹兼顾。

　　五是必须着力构筑“沿江、沿路”两大战略载体。按照“三集中”原则，科学规划好产业发展空间、城镇发展空间、群众居住生活空间和生态保护空间。重点构筑好沿江基础产业带和沿沪宁高速公路高新技术产业带，提高产业集中度，使镇江制造业实现组团式发展、规模化推进、特色性提升。

　　三、奋力做好2006年工作

　　2006年是“十一五”发展的起始之年，做好今年的政府工作至关重要。必须认真落实市委四届十二次全会的要求，坚持以率先为基调，以统筹为原则，以富民为目标，以创新为动力，以和谐为追求，确保更快更好发展。

　　全市国民经济和社会发展的主要预期目标为：地区生产总值增长13.5％，单位生产总值能耗下降4％；全社会固定资产投资增长20％；财政总收入增长15％，地方财政一般预算收入增长15％；工商注册实际到位外资增长20％，外贸出口增长18％；社会消费品零售总额增长14％；城镇居民人均可支配收入增长12％，农民人均纯收入增长9％；城镇登记失业率控制在4％以内。工作中，我们将力争做得更好。

　　实现上述目标，我们要突出抓好六个方面工作：

　　（一）加快富民惠民步伐

　　积极扶持创业。全面落实有关奖励减免、小额贷款等促进政策，改善创业环境，降低创业门槛，鼓励自主创业。提高资产性、经营性收入占居民收入的比重，壮大中等收入群体。今年全市财政共安排农业创业担保基金2000万元。

　　努力扩大就业。建立城乡统筹的就业服务和培训网络，加强职业技能培训。全市财政共安排专项资金600万元，支持农村劳动力培训。鼓励非公有制经济创造更多就业岗位，大力开发社区就业和公益性就业岗位。完善企业工资指导线制度，严格执行最低工资标准，建立企业工资增长的内生机制。妥善分流安置改组改制、破产企业职工。严禁拖欠农民工工资，保障农民工合法权益。

　　健全保障体系。建立扩面征缴长效机制，提高个体私营等非公有制企业的社会保险参保率。完善灵活就业人员的参保办法，妥善解决关闭破产企业退休人员的医疗保障问题。健全城乡最低生活保障制度，形成低保标准随经济发展适时增长的机制，完善和落实被征地农民基本生活保障制度。以市慈善总会为依托，健全社会救助体系，广泛开展扶贫帮困活动。对困难群体实施分类救助。加强法律援助，维护弱势群体合法权益。

　　（二）加快建设社会主义新农村

　　按照中央建设社会主义新农村的“二十字”目标，围绕“先行试点、典型引导、重点突破、初见成效”的要求，努力做好农村工作。今年，各辖市（区）都要明确1－2个新农村建设的试点。

　　加快农业结构调整。优化农业生产布局，推进农业产业化经营。按照规模、高效的原则，全年实施农业结构调整项目100个。加快推进农业标准化和信息化建设，健全农业技术推广、农产品市场、农产品质量安全和动植物病虫害防控体系，提高农业机械化水平。加大农业龙头企业的培育和扶持力度，新增农业龙头企业10个以上。大力发展农民专业合作社和农民经纪人队伍。加大信贷支农力度，鼓励“三资”开发农业。全市财政共安排2000万元，推进丘陵山区农业综合开发和茅山老区开发。

　　改善为农服务。鼓励连锁经营等新型商贸业态往农村发展，开拓农村市场，完善农村流通体系，提高农村消费水平。引导社会中介服务组织进村入乡，使农民享受到更多的生活便利。

　　推进农村基础设施建设。启动新一轮农村道路规划建设，推进农村供水、环保、电力、通讯、水利等基础设施建设。继续实施农村“双清双美”，疏浚县乡河道42条，改善农村卫生状况和村容村貌。

　　促进乡风文明、管理民主。加强乡镇文体活动场所建设，大力开展移风易俗活动和“三下乡”活动，丰富农村业余文化生活。倡导用村规民约实施自我约束、自我管理，强化村务公开，体现群众意志。

　　（三）加快经济结构调整

　　提升工业发展层次。着力调高工业结构，推进工业向高端化、高层次发展。一是推进自主创新。企业是自主创新的主体，政府要加大引导和扶持力度，激活企业自主创新的内在动力。今年市财政安排科技成果转化专项扶持资金1000万元，辖市（区）财政共安排4000万元。结合镇江实际，把增强引进消化吸收再创新的能力，作为自主创新的重点，努力使镇江的自主创新能够走在前列。抓好生产力促进中心、镇江新区留学生创业园等平台建设，建立以市场为导向、产学研结合的企业技术创新体系。全年新增企业技术中心10家，新培育省级以上高新技术企业超过20家，认定省级高新技术产品60项以上。二是努力壮大规模。围绕主导产业、优势产业，做大核心企业，做强主导产品。努力使销售超亿元企业达到240家，10亿元企业达到20家，力争培育1家百亿元企业。继续实施大项目战略，重点抓好索普甲醇、大亚木业二期、鼎胜铝制品等一批在手在建的大项目。全年完成工业性投入280亿元以上。三是实施品牌发展战略。引导企业以技术和效益为核心，培育在国内外有较高知名度的名牌产品。全年新增全国驰名商标1个、中国名牌2－3个、国家免检产品2－3个。

　　提升服务业发展水平。优先发展旅游业。实施旅游业发展三年行动计划，继续推进“三山”、南山风景区和世业洲旅游度假区建设，加快开发九龙山、西津渡古街区等旅游文化资源。进一步提升商贸流通业发展水平。加强重点市场、大型超市和特色街区建设，发展连锁经营、物流配送、电子商务等新型业态，依托港口、重点开发园区和大型商贸市场，加快综合物流园区和专业物流中心建设。重点打造以大港三期为依托的口岸物流、以润扬国际工业物流中心为依托的第三方物流、以特色产业集群为依托的大型专业市场。促进房地产业健康发展，活跃住房二级市场和租赁市场。继续推进保险、信息、中介服务业和文体产业加快发展，鼓励社会消费多元化。

　　深化体制机制改革。完善国有资产监管体制改革和产权制度改革，加快建立现代企业制度，健全法人治理结构。营造公平竞争环境，鼓励民营企业做优做强。探索组建服务于民营中小企业的金融、技术、管理类组织，努力解决好制约民营经济发展的瓶颈。稳健推进生产经营性事业单位转企改制工作。深化财政改革，巩固和完善预算编制、执行、监督三分离的运行机制，进一步理顺市、区财政运行体制。加强金融改革与创新，支持地方经济发展。

　　（四）加快提高对外开放水平

　　全力推进招商引资。全年争取引进超亿美元项目8个。重点引进机电、化工、新材料等产业项目，优先引进科技含量高、吸纳就业多、环境污染少的项目，鼓励“零土地”招商和外商增资扩股。抢抓服务业扩大对外开放新机遇，力求服务业引资取得新突破。改进招商方式，降低招商成本，强化招商队伍建设，提高项目服务水平。

　　突出沿江开发和园区建设。镇江新区争取成为国家级开发区，筹建公共型保税仓库。引导各省级经济开发区进一步明确发展方向，提高规划水平，加大资源整合力度，加快基础设施建设，增强承载能力，提升县区利用外资在全省的位次。加快“大通关”建设，提高服务开放型经济的水平。加大港口投入，新建泊位11个，其中万吨级7个；竣工泊位11个，其中万吨级3个。完成港口吞吐量6000万吨、口岸外贸运量1200万吨、集装箱运量20万标箱。

　　努力扩大外贸外经。推动特色工业品和农副产品采用国际标准，进入国际市场，扩大出口总量。继续实施“走出去”战略，支持资源开发类企业到境外开展合资合作，鼓励企业开展境外加工贸易；做好“窗口型”外经公司注册登记和外经经营权申报工作，扩大对外工程承包，以境外工程带动劳务输出，提升劳务经济层次。进一步做好外事、侨务和对台工作。

　　（五）加快城市建设步伐

　　推进南徐新城建设，重点做好起步区基础设施建设，启动文化体育会展中心和市行政服务中心前期工作，开工建设市规划展示中心。推进北部滨水区建设，重点抓好引航道节制闸、金山湖退渔还湖、清淤造景等基础工程建设。

　　继续完善城市基础设施。在对外交通体系上，建设镇溧、宁常高速公路和宁杭高速公路镇江段二期工程，实施大港至丹阳、镇江至禄口机场通道等干线工程，做好镇泰过江通道建设准备工作。在市内交通网络上，完成花山东路、天桥路拓宽等一批主次干道建设。优先发展城市公交，提升智能化管理水平，增强节点通行能力。继续加大公共领域投入力度，实施大港、高资供水工程和市区排涝系统改造。加快停车设施规划、改造和建设。加大电网建设力度，建成500千伏、220千伏等一批输变电工程。抓好城市环境综合整治，严禁违法用地和非法建设行为。

　　提升生态建设水平。建成市级以上环境优美乡镇4个、生态村10个。加快城市森林生态体系规划建设，实施山体保护和美化绿化工程，继续开展限制开山采石专项整治，加大采石宕口整治复绿力度。推进沪宁高速、312国道两侧绿化景观工程和街头小游园建设。抓好水环境综合治理和污水处理系统主干网工程建设。严格控制污染物排放总量，实现工业污染源稳定达标排放，强化城市噪音、扬尘治理和汽车尾气控制。积极推行清洁生产，加强资源综合利用，发展循环经济，提倡绿色消费，建设资源节约型、环境友好型社会。

　　（六）加快建设和谐社会

　　高度重视精神文明建设。以争创全国文明城市为龙头，整体推进文明行业、文明机关、文明社区、文明村镇创建活动。从建立个人和企业信用入手，加快构建社会信用体系。军民共建和谐镇江，争创全国双拥模范城“五连冠”。全面推进“现代文明市民教育工程”，大力弘扬新时期“镇江精神”，让“创业、创新、创优”成为镇江人的自觉追求。

　　协调发展社会事业。抓好“火炬”、“星火”计划等各类科技计划项目的落实。完善农村义务教育管理体制，促进义务教育优质化、均衡化发展，推进区域教育现代化。建设市十一中新校区和丁卯片区配套初中。努力办好地方高等学校。加大高层次和紧缺专业人才的引进培养力度，新增高层次人才1500人。积极发展职业技术教育，新增高级技工2500人、技师500人。强化政府公共卫生管理职能，加大对医疗秩序、医疗市场和违法行医、违规药品广告的治理力度，规范医疗机构服务行为，着力解决群众看病难、看病贵的问题。加强农村卫生服务体系建设，新型农村合作医疗覆盖率继续保持在90％以上。进一步完善疾病防控体系，做好重大传染病防治工作，保障公共卫生安全。切实做好防震减灾工作。加快人口与计划生育新机制建设。加大政府对公益性文化事业的投入，以纪念镇江成为国家历史文化名城20周年为契机，进一步做好历史文化遗产的保护和利用工作。活跃广场文化、社区文化和村镇文化，繁荣文艺创作。扩大群众性体育普及面，提高竞技体育水平，力争十六届省运会上金牌总数、团体总分双超上届。注重发挥工会作用，依法维护企业职工和进城务工人员的合法权益。支持共青团、妇联等群众团体的工作。

　　切实维护社会稳定。坚决纠正各种损害群众合法利益的行为，促进社会利益关系的和谐。加强社会治安综合治理，依法严厉打击各类犯罪活动，巩固“平安镇江”创建成果，建设“法治镇江”。建立健全公共突发事件应急机制，完善指挥和救援体系，提高维护公共安全和社会稳定能力。严格落实安全生产责任制和责任追究制，预防和减少重大事故的发生。深入开展法制宣传教育。进一步加强信访工作，健全“大调解”机制，及时处置各类群体事件，确保社会安定、环境安全、百姓安宁。

　　今年，我们将为人民群众办好15件实事：1、新增城镇就业3万人，转移农村劳动力5万人；2、加强市行政服务中心建设，设立行政服务便民窗口，建立市、辖市、乡镇（街道）便民服务网络；3、完成市区40万平方米老住宅小区和2万平方米街巷道路改造，继续整治城区低洼积水区，对部分老住宅小区和街巷进行绿化配套；4、新建定建房5万平方米，改造危房1万平方米；5、按计划实施京岘山路和花山湾二区两处山体滑坡综合治理；6、市区新增50辆环保节能公交车，新辟和调整优化5－6条公交线路；7、淘汰4蒸吨以下燃煤锅炉及生活大灶100台左右；8、建成镇江农副产品批发市场一期工程；9、建立全市农业信息化支撑体系，为农村居民提供网上服务；10、新建农村敬老院5所，改、扩建23所；11、新建社区卫生服务中心（站）16个，街道（乡镇）社区卫生服务中心规划建设率达到100％，实施“人人享有医疗保障”工程；12、新增农村无害化户厕7.25万户，全市农村无害化户厕普及率达50％以上；13、新建全民健身工程（点）50个；14、免除城乡低保家庭在校生义务教育杂费等6项费用；15、易地新建市旅游学校，建成市青少年校外活动中心。

　　各位代表，新形势、新任务对政府工作提出了更高要求，我们将进一步围绕“创新、服务、高效、规范、清廉”的目标，精心打造顺应发展形势、体现人民意志的现代政府。

　　努力建设责任政府。只有负责任的政府，才是人民群众最信赖的政府。要建立和严格执行行政问责制、责任追究制，重点对政府部门行政首长在决策失误、执行不力、不认真履行职责、内部监管不到位等方面进行问责。做到有权必有责、有责必问责、有错必追究，提高政府执行力和公信力。

　　努力建设法治政府。法律，是政府一切工作的准绳。要严格依法执政，确保政府各部门及其工作人员按照法定权限和程序行使职权、履行职责。依法完善行政管理决策机制。对涉及经济社会发展全局的重大事项，健全协商和协调机制；对专业性、技术性较强的重大事项，健全专家论证、技术咨询、决策评估机制；对与群众利益密切相关的重大事项，健全公示、听证制度。

　　努力建设高效政府。要视效率为政府的生命。进一步减少和规范行政审批，确保市行政服务中心“两集中、两到位”全部推进到位，并切实提高办结时效。每一名政府工作人员都要强化服务意识，放宽视野谋发展，埋头苦干抓落实，努力以政府的高效运作，促进镇江的快速发展。

　　努力建设廉洁政府。深入推进反腐倡廉工作，建立健全有利于从源头上防范腐败的体制机制。加强经济责任审计和行政监察工作。继续推行政务公开，今年起实施政府新闻发布制度。自觉接受市人大及其常委会的监督，接受人民政协的民主监督，接受新闻舆论和社会公众监督。努力建设一支政治坚定、勤于学习、业务精通、清正廉洁、作风优良的公务员队伍。

　　各位代表，形势催人奋进，镇江前程似锦！让我们在中共镇江市委的坚强领导下，紧紧依靠全市人民，为建设一个生机勃勃、充满希望的新镇江而努力奋斗！
